# Routine health information system utilization and factors associated thereof among health workers at government health institutions in East Gojjam Zone, Northwest Ethiopia

**DOI:** 10.1186/s12911-017-0509-2

**Published:** 2017-08-07

**Authors:** Atsede Mazengia Shiferaw, Dessalegn Tegabu Zegeye, Solomon Assefa, Melaku Kindie Yenit

**Affiliations:** 1 Department of Health Informatics, University of Gondar, P.o.box: 196 Gondar, Ethiopia; 2 Department of Epidemiology and Biostatistics, University of Gondar, Gondar, Ethiopia

**Keywords:** Routine health information utilization, Health workers, Northwest Ethiopia

## Abstract

**Background:**

Using reliable information from routine health information systems over time is an important aid to improving health outcomes, tackling disparities, enhancing efficiency, and encouraging innovation. In Ethiopia, routine health information utilization for enhancing performance is poor among health workers, especially at the peripheral levels of health facilities. Therefore, this study aimed to assess routine health information system utilization and associated factors among health workers at government health institutions in East Gojjam Zone, Northwest Ethiopia.

**Methods:**

An institution based cross-sectional study was conducted at government health institutions of East Gojjam Zone, Northwest Ethiopia from April to May, 2013. A total of 668 health workers were selected from government health institutions, using the cluster sampling technique. Data collected using a standard structured and self-administered questionnaire and an observational checklist were cleaned, coded, and entered into Epi-info version 3.5.3, and transferred into SPSS version 20 for further statistical analysis. Variables with a *p*-value of less than 0.05 at multiple logistic regression analysis were considered statistically significant factors for the utilization of routine health information systems.

**Results:**

The study revealed that 45.8% of the health workers had a good level of routine health information utilization. HMIS training [AOR = 2.72, 95% CI: 1.60, 4.62], good data analysis skills [AOR = 6.40, 95%CI: 3.93, 10.37], supervision [AOR = 2.60, 95% CI: 1.42, 4.75], regular feedback [AOR = 2.20, 95% CI: 1.38, 3.51], and favorable attitude towards health information utilization [AOR = 2.85, 95% CI: 1.78, 4.54] were found significantly associated with a good level of routine health information utilization.

**Conclusion:**

More than half of the health workers working at government health institutions of East Gojjam were poor health information users compared with the findings of others studies. HMIS training, data analysis skills, supervision, regular feedback, and favorable attitude were factors related to routine health information system utilization. Therefore, a comprehensive training, supportive supervision, and regular feedback are highly recommended for improving routine health information utilization among health workers at government health facilities.

## Background

Public health decision-making is seriously reliant on a timely availability of sound data, and globally significant human and financial resources have been invested to improve health information systems [1, 2]. Despite the fact that data monitoring and evaluation has been improved through information systems, data demand and information use for decisions have often been negligible among stakeholders. As a result, many health systems fail to fully link evidence to decisions and suffer from inadequate ability to respond to priority health needs at all levels of the health system. It is well accepted that information generated by health care systems is used for planning, management of health commodities, detecting outbreaks, and monitoring the overall performance of the health system that further maintains the quality of care [3, 4].

Routine health information system (RHIS) is the backbone for planning and management of health services at district levels as it can play an important role in effective and efficient health service delivery, decision making, and the improvement of the program [3–6]. Health Information System (HIS) is a system that integrates data collection, processing, reporting, and use of the information necessary for improving health service effectiveness and efficiency through better management at all levels of health services. Health management information system (HMIS) is a means that allows collection and storage as well as analysis and usage of health data that assist decision makers and stake-holders to manage and plan resources at all levels. It is also used to improve patient satisfaction with health services by tracking certain dimensions of service quality. However, the value of health information is determined by its utilization in decision-making; data generated at peripheral levels of the health system usually put in reports and shelves are not sufficiently utilized to improve health care [5–9]. Most health workers in developing countries associate information system with filling endless registers by names and addresses of patients, compiling information on disease every week or month, and sending reports to the next level without adequate utilization and feedback [10–13].

Effective data analysis, interpretation, and utilization of information at all levels of the health system is very important for evidence based decisions [2]. Though, in resource limited countries where evidence based decision through better information system is highly required, routine health information system utilization is low [9, 10, 14, 15]. In Africa, the level of health information utilization has been poor, ranging from 10 to 56% [16–22]. In Ethiopia, information quality and use remain weak within the health sector, particularly at the peripheral levels of districts and health facilities which have primary responsibilities for operational management [23]. As a result, most managerial decisions are being made without evidence, resulting in the failure of many health programs, perhaps because the information system in the country is cumbersome and fragmented. According to the 2002 review, health information systems at district health facility levels are weak, leading to poor quality data reporting to the next level without feedback and use [24]. Literature shows that routine health information utilization can be affected by organizational factors [25] and technical and behavioral characteristics of health workers [2]. Among the influential factors, health workers’ data analysis skills [21, 26, 27], regular supervision, and feedback [21, 27, 28] are markedly associated with routine health information utilization.

Evidence based decision making through health information system utilization has become the top priority on the agenda of the government of Ethiopia and its development partners. Ethiopia has been strongly committed to strengthen the national Health Information System (HIS) through HMIS, and monitoring and evaluating (M&E) performance. Thus, the policy has identified Health Management Information System (HMIS) as a key component for a successful implementation of the Health Sector Development Program (HSDP) strategic plan. Ever since the implementation of HMIS in 2008, the Ministry of Health has distributed guidelines, built capacity on health data, and established a standardized and integrated data collection and reporting formats. In addition, the use of information, and appropriate technology has been considered as critical factors for strengthening and improving health sector management information system (HMIS) [23]. Recently, the National Health Information Revolution Road Map introduced Community Health Information System (CHIS) to capture basic health and health related information at household and individual levels. Similarly, Electronic Health Record (EHR) has been established at all levels of the health system to improve patient care and to provide accurate data for informed decision making. In the Amhara Region, where the study was conducted, health information utilization in district health offices has been partial and inconsistent [24]. To be sure, data on routine health information system utilization was limited in the study setting, and the inadequacy of information use among health workers remains a problem. Hence, the study assessed routine health information utilization among health workers in East Gojjam Zone government health facilities.

## Methods

### Study design and setting

An institution-based cross-sectional study was conducted from April to May, 2013, in government health facilities of East Gojjam Zone. The capital of the zone (Debremarkos) is located 300 km from Addis Ababa, the capital of Ethiopia. According to the Plan and Programs report of the zonal health department, there were 4876 professionals in the two hospitals, 18 health offices, and 91 primary health units (health centers and health posts) of the zone.

### Study participants, sample size, and sampling procedure

All health workers in the selected health facilities were included in the study. Sample size was calculated using the single population proportion formula, considering the following assumptions: 33% prevalence of health information utilization at a district level in Jimma [22], 95% level of confidence, 5% of margin of error, a design effect of 2, and 5% of non-response rate. Finally, the minimum sample size of 668 was obtained. In the zone, there were two hospitals and 91 primary health units (health centers and health posts). Out of the total health facilities, one hospital, 27 health centers, and health posts (PHU) were selected by the simple random sampling technique.

### Data collection tool and procedure

The questionnaire was adopted from PRISM framework assessment tool version 3.1 [2, 29]. The tool collects detailed information on Health Information System (HIS) as its input, process and output, and indicates the major factors affecting the performance of a routine health information system. In its conceptual framework, behavioral, technical, and organizational factors are the major determinants of the utilization of routine health information systems. Among the behavioral factors, knowledge, skills, attitudes, and motivation of the people who collect and use data, and technical factors such as data collection processes, systems, and methods, as well as organizational and environmental determinants, like information culture, structure, resources, roles, and responsibilities of the health system affect the utilization of routine health information systems.

The tool which was prepared in English was translated to Amharic (the native language of the study) and retranslated to English by a professional translator and a public health expert. Data were collected using the pretested structured self-administered questionnaire by means of an observational checklist. Seven health professionals who had HMIS training and prior data collection experience were assigned for data collection. Two health professionals who had experience in HIS monitoring supervised the task. Two days’ intensive training was given to data collectors and supervisors on the objective of the study and the confidentiality of information. Awareness was created on the objective of the study, and respondents were informed that their response would not affect the possible work efficiency score they needed for promotion.

### Operational definitions and study variables

The outcome variable, routine health information system utilization was measured using the Performance of Routine Information System Management (PRISM) assessment tool. Thus, it was defined as the use of such information for:

- the day-to-day management of health service facilities and districts;
- displaying data for monitoring the key objectives of health services and showing key indicators by means of graphs and tables;
- finding out whether the health professionals can gather data to detect the causes of health problems to prioritize the problems and use the data for health education;
- using the data to identify and manage epidemics; and
- Observing the trends of health services and using the data for drug supply and management.

All these components adapted from the PRISM assessment tool have a likert scale measure, ranging from “strongly disagree” to “strongly agree”. Finally health workers’ mean scores were used to split health professionals’ health information utilization into “has good routine health information practice”, or “has poor routine health information utilization practice” practices. In this study, health workers (doctors, health officers, nurses, laboratory personnel, health extension workers, etc.) are defined as any health personnel who are collecting health data in order to utilize health information for the improvement of health status.

### Data processing and analysis

Data were entered into Epi-info version 3.5.3 and exported to a statistical package for social science (SPSS) version 20 for further analysis. Descriptive statistics, including frequencies and proportions were computed using the binary logistic regression model in order to summarize the variables. To control the possible effects of confounders, variables with a *p*-value of less than 0.2 in the bi-variable analysis were entered into the multivariable logistic regression analysis. Both Crude Odds Ratio (COR) and Adjusted Odds Ratio (AOR) with 95% confidence intervals were computed to show the strengths of associations. The technique was a backward stepwise regression method. Finally, a *p*-value of less than 0.05 at the multivariable logistic regression analysis was used to identify variables significantly associated with the utilization of a routine health information system. For this study, Hosmer and Lemeshow’s goodness of fit test which gave a large *p*-value was considered.

## Results

### Socio-demographic characteristics

A total of 635 health workers were included in the study, with a response rate of 94.8%. Nearly two-thirds (62%) of the respondents were females. A bit higher than half (51.9%) of the respondents were diploma graduates. The majority of the respondents in this study (41.4 and 29.8%, respectively) were nurses and health extension workers (Table 1).

**Table 1:** Socio-demographic and economic characteristics of respondents working at government health facilities at East Gojjam Districts, Northwest Ethiopia, 2013

Variables	Frequency (#)	Frequency (%)
Sex		
Female	394	62.0
Male	241	38.0
Age (years)		
20–24	207	32.6
25–29	301	47.4
30–34	78	12.3
35–39	21	3.3
40–44	12	1.9
≥ 45	16	2.5
Monthly salary (ETB)		
700–1200	136	21.4
1201–1600	278	43.8
1601–200	98	15.4
2001–2400	36	5.7
2401–2800	43	6.8
≥ 2800	44	7.0
Service in year		
< 5	369	58.1
5–10	224	35.3
≥ 11	42	6.0
Educational status		
Certificate	187	29.4
Diploma	329	51.9
Degree and above	119	18.7
Field of Study		
Medical doctor	8	1.3
Health officer	49	7.7
Nurse	263	41.4
Health extension	189	29.8
Laboratory personnel	39	6.1
Pharmacy	31	4.9
Midwifery	33	5.2
Others	12	1.9

### Organizational and technical factors

While more than two-thirds (68.2%) of the respondents were not supervised, surprisingly almost half (53.3%) of them were supervised every 6 months. More than half (53.5%) of the respondents did not receive any regular feedback from the next higher health authority. Regular feedback communication was better, 45.5 and 51.8%, at health centers and health posts, respectively, than the 40.8% at hospitals. One-fifth (22.0%) of the respondents did not assume that the feedback given was not relevant to improve health information utilization. It was found that equipment for data analysis such as computers were not available in most (93.4%) of the health departments/units. When it comes to the availability of HMIS resources at health facilities, only 6.6% of the departments had computers, while 5.7 and 0.8%, respectively, owned printers and the internet. With respect to HMIS infrastructure, one-third (35.0%) of the respondents reported that no professional was assigned for health information system in their health facility. Similarly, almost one-third (65.0%) of the respondents said that there was no standardized registration book for their day-to-day activities. On the other hand, almost half (51.0%) of the respondents indicated that they faced shortage of reporting formats during reporting (Table 2).

**Table 2:** Organizational characteristics of government health facilities in East Gojjam zone, Northwest Ethiopia, 2013

Variables	Frequency (#)	Percentage (%)
Have supervision		
Yes	433	68.2
No	202	31.8
Frequency of supervision		
Every month	89	20.5
Every 3 month	39	9.0
Every 6 month	231	53.3
Every year	74	17.2
Have received regular feedback		
Yes	295	46.5
No	340	53.5
Relevance of feedback		
Yes	265	78.0
No	75	22.0
Regular feedback at Health posts		
Yes	102	51.8
No	95	48.2
Regular feedback at Health centers		
Yes	140	45.5
No	168	54.5
Regular feedback at Hospital		
Yes	53	40.8
No	77	59.2
Computer in the department		
Yes	41	6.6
No	583	93.4
Printer in the department		
Yes	36	5.7
Telephone in the department		
Yes	9	1.4
Fax in the department		
Yes	4	0.6
Internet in the department		
Yes	5	0.8
Personnel assigned to Health Information system		
Yes	413	65.0
No	222	35.0
Have separate room for Health Information System		
Yes	258	40.9
No	372	59.1
Specific budget assigned for Health Information System		
Yes	139	22.1
No	491	77.9
Standardized registration book		
Available	413	65.0
Not available	222	35.0
Reference material in the department		
Available	390	61.9
Not available	240	38.1
Standardized reporting formats		
Available	473	74.9
Not available	158	24.1
Shortage of reporting formats		
Yes	324	51.0
No	311	49.0

Regarding technical factors, the majority (92.4 and 95.0%, respectively) of the respondents received no training on basic computer skills and data analysis and management. More than half (53.2%) of the respondents did not receive training on health management information system (HMIS). Data analysis skills training were given to 5% of the health workers. Similarly, training on planning and basic computer skills were given to 28.9 and 7.6% of the health workers, respectively. Training needs of health workers were assessed, and it was reported that the three most needed trainings were HMIS, basic computer, and data analysis skills. Almost half, (49.9%), of the respondents heard of the role of health information utilization at district health facility levels. Only about one-third (36.7%) of the respondents reported that they studied about health information utilization through trainings (Table 3).

**Table 3:** Technical characteristics of government health facilities in East Gojjam zone, Northwest Ethiopia, 2013

Variables	Frequency (#)	Percentage (%)
Training on basic computer skill		
Yes	48	7.6
No	587	92.4
Training on planning		
Yes	152	28.9
No	483	71.1
Training on Health Management Information System		
Yes	338	53.2
No	297	46.8
Training on data analysis and management		
Yes	32	5.0
No	603	95.0
Training on data utilization and interpretation		
Yes	47	7.4
No	588	92.6
Heard health information utilization		
Yes	317	49.9
Source of information		
Training	117	36.7
Friends	96	30.2
Electronic media	70	22.1
Others sources	34	10.7

### Routine health information system utilization

In this study, good routine health information system utilization was noted among 45.8% of the health workers. The proportion of good health information utilization was 51.3% at primary health care units, 42.1% at health posts, and the least (38.5%) at hospitals.

### Factors associated with good routine health information system utilization

In the bivariable logistic regression analysis, knowledge on health information use, favorable attitude, daily documentation, computer and data analysis skills, supervision, regular feedback, HMIS training, presence of HMIS personnel in the health facility, and computers in departments were factors associated with good routine health information utilization at a *p*-value of less than 0.2. Consequently, these variables were subjected to the multivariable logistic regression analysis, and it was noted that data analysis skills, supportive supervision, regular feedback, favorable attitude, and type of health facility were significantly associated with good routine health information utilization at a *p*-value of 0.05.

In this study, higher odds of good routine health information system utilization were noted among health workers who had good data analysis skills [AOR = 6.40; 95% CI: 3.93, 10.37], supportive supervision [AOR = 2.60; 95% CI: 1.42, 4.75], regular feedback [AOR = 2.20; 95% CI: 1.38, 3.51], and HMIS training [AOR = 2.72; 95% CI: 1.60,2 4.6]. Similarly, increased odds of good health information use were observed among health professionals who had favorable attitude towards the use [AOR = 2.85; 95% CI: 1.78, 4.54] and those who were working at hospitals [AOR = 2.35; 95% CI: 1.48, 4.90] (Table 4).

**Table 4:** Factors associated with routine health information utilization among health workers in government health facilities in East Gojjam zone, Northwest, 2013

Variable	Routine health information Utilization		OR (95% CI)	
	Poor	Good	Crude	Adjusted
Type of facility				
Health post	83 (42.1%)	114 (57.9%)	0.69 (0.65,0.73	0.34 (0.322,6.05)
Health center	158 (51.3%)	150 (48.7%)	1	1
Hospital	50 (38.5%)	80 (61.5%)	1.69 (1.11,2.56)	2.35 (1.48,4.90)*
Supervision				
Yes	267 (61.7%)	166 (38.3%)	1	1
No	24 (11.9%)	178 (88.1%)	11.93 (7.47,19.1)	2.60 (1.42,4.75)*
Regular feedback				
Yes	209 (70.8%)	86 (29.2%)	1	1
No	82 (24.1%)	258 (75.9%)	7.65 (5.37,10.89)	2.20 (1.38,3.51)*
HMIS training				
Yes	200 (67.3%)	97 (32.7%)	1	1
No	91 (26.9%)	247 (73.1%)	5.60 (3.40,7.87)	2.72 (1.60,4.62)*
Basic computer training				
Yes	37 (77.1%)	11 (22.9%)	1	1
No	254 (43.3%)	333 (56.7%)	4.41 (2.21,8.82)	2.06 (0.79,5.39)
HMIS personnel in the facility				
Yes	155 (59.8%)	104 (40.2%)	1	1
No	136 (36.2%)	240 (63.8%)	2.63 (1.90,3.64)	1.013 (0.53,1.93)
Have computer in the department				
Yes	26 (57.8%)	19 (42.2%)	1	1
No	265 (55.1%)	325 (44.9%)	1.68 (0.91,3.10)	1.094 (0.44,2.72)
Computer skill				
Yes	90 (76.3%)	28 (23.7%)	1	1
No	201 (38.9%)	316 (61.1%)	5.05 (3.19,8.00)	1.20 (0.58,2.50)
Data analysis skill				
High	240 (73.4%)	87 (26.6%)	1	1
Low	51 (16.6%)	257 (83.4%)	13.90 (9.43,20.4)	6.40 (3.93,10.37)*

* Significant at a *p*-value of < 0.05

## Discussion

The Ethiopian Federal Ministry of Health emphasized HMIS as a key to a successful implementation of the Health Sectors Transformation Plan (HSTP) and achieving the Sustainable Development Goals. Considering this initiative, the Ethiopian Health Sector Strategic Plan underlined that routine data generated at district health facilities should be considered as the entrance to utilizing health information and a primary source of information for continuous monitoring of health services in the country, and that data should be utilized at the place where it was generated [30]. This study aimed to assess routine health information utilization and its associated factors in East Gojjam zone government health facilities.

In this study, 45.8% of health workers demonstrated a good level of routine health information utilization. The finding was more than those of Jimma and Arisi, Ethiopia, which were 32.9 and 32.1%, respectively [22, 31]. This variation might be due to the presence of HMIS personnel and units in our study area compared to the other sites [22, 31]. Reports also revealed that effective and efficient HMIS is critical for health care information system in that it provides data for planning, setting of targets, and implementation [32, 33]. In contrast, the level of routine health information utilization proportion in our study was lower than those of studies reported from outside Ethiopia, Uganda (59%) [17], and South Africa, (65%) [18]. This might be due to the difference in health information system structures and health professional attitude for routine health information system [5, 34]. Reports also showed that strengthening health information system focusing on technical, behavioral, and organizational structures is one essential components for improving the quality and use of data for decision making at all levels of the health system [35, 36].

Out of the variables which showed significant association with routine health information system utilization, higher odds were noted among health workers who had good data analysis skills compared to workers who had poor skills. The finding was supported by those of other studies reported elsewhere [5, 21, 22, 37]. This might be due to the skills of health workers to transform routine data into meaningful information. A study conducted in India underlined that even though health information utilization depends on data analysis skills, organizational factors play a great role in exercising the skills [38].

Like other studies conducted elsewhere [21, 31], the odds of routine health information system utilization in this study was higher among health workers who had training on HMIS. This might be due to the fact that health professionals who trained on HMIS had the potential to compile, analyze, and utilize information generated in the routine day-to-day activities. However, studies in Tanzania and Uganda showed that HMIS had no significant association with health information utilization. This might be due to the shortage of personnel trained in HMIS in the areas studied in Tanzania (19%) [39] and Uganda (9%) [15] compared with ours (46.8%).

The odds of health information system utilization were lower among health workers at hospitals when compared with those at primary health care units (health centers, health posts). This might be due to the great attention paid by the government to district health facilities by providing supervision and regular feedback. In accordance with this justification, this study noted that health workers who had regular feedback had 2.2 times higher initiative to utilize routine health information system when compared with health workers who had no feedback. Health workers who receive regular feedback on their report might receive constructive and relevant advice to utilize their data for improving their service delivery [40]. Reports also showed that regular feedback given to health care providers is an essential component of any reporting system to improve the service and utilization of information systems [41, 42].

Furthermore, the odds of health information utilization among health workers who had supervision were higher than those of their counterparts. This might be due to the fact that supervision has a significant role in identifying the gaps and improving health workers’ performance. One study also reported that supervision, usually at quarterly intervals by most programs, is identified as an essential element for improving the overall performance, particularly the quality of care [22, 28].

The study attempted to show the level and the predictors of routine health information system utilization, particularly among health professionals. However, the study was not free from limitations, such as inability to include qualitative methods to measure health professional’s culture of health information utilization and other organizational factors. In addition, the cross-sectional design might have prevented the work from showing temporal relationships. Besides, the study was not able to include health professionals in private institutions.

## Conclusion

This study concluded that more than half of the health workers in East Gojjam government health facilities had poor routine health information system utilization. HMIS training, data analysis skills, supervision, regular feedback, and type of health facility were found to have significant associations with routine health information system utilization. Therefore, training on HMIS, strengthening supervision and regular feedback at health facilities are highly recommended. Furthermore, further research is suggested for assessing health workers’ culture of health information utilization at the lower health facilities where data are generated.

## Abbreviations

AOR: Adjusted odds ratio
CI: Confidence interval
HMIS: Health Information System
PHU: Primary Health Unit
PRISM: Performance of Routine Information System Management
SPSS: Statistical Package for Social Science
